# Estrogen Signaling in Metabolic Inflammation

**DOI:** 10.1155/2014/615917

**Published:** 2014-10-23

**Authors:** Rosário Monteiro, Diana Teixeira, Conceição Calhau

**Affiliations:** ^1^Department of Biochemistry, Faculty of Medicine, University of Porto, Medical Investigation Center, 4200-319 Porto, Portugal; ^2^Center for Research in Health Technologies and Information Systems (CINTESIS), 4200-450 Porto, Portugal

## Abstract

There is extensive evidence supporting the interference of inflammatory activation with metabolism. Obesity, mainly visceral obesity, is associated with a low-grade inflammatory state, triggered by metabolic surplus where specialized metabolic cells such as adipocytes activate cellular stress initiating and sustaining the inflammatory program. The increasing prevalence of obesity, resulting in increased cardiometabolic risk and precipitating illness such as cardiovascular disease, type 2 diabetes, fatty liver, cirrhosis, and certain types of cancer, constitutes a good example of this association. The metabolic actions of estrogens have been studied extensively and there is also accumulating evidence that estrogens influence immune processes. However, the connection between these two fields of estrogen actions has been underacknowledged since little attention has been drawn towards the possible action of estrogens on the modulation of metabolism through their anti-inflammatory properties. In the present paper, we summarize knowledge on the modification inflammatory processes by estrogens with impact on metabolism and highlight major research questions on the field. Understanding the regulation of metabolic inflammation by estrogens may provide the basis for the development of therapeutic strategies to the management of metabolic dysfunctions.

## 1. Introduction

The epidemic increase in obesity has been paralleled by the rise in cardiovascular disease, type 2 diabetes, fatty liver, cirrhosis, asthma, neurodegenerative diseases, and certain types of cancer [1, 2] and subsequently linked to reduced life expectancy and premature death [3].

The finding that obesity is associated with a systemic low-grade subacute inflammatory state and that inflammation mediates many of the pathological consequences of obesity has opened the avenue to the study of the interactions between metabolic and immunological processes [2]. After the acknowledgment of the adipose tissue (AT) as a true endocrine organ secreting hormones, cytokines, chemokines, and growth factors with influence on metabolism, vascular function, appetite and satiety, and fertility, among many others, came its recognition as a fundamental player in innate and acquired immune processes [4]. AT dysfunction has been considered a central component of obesity-related inflammation and the main instigator of the pathological consequences of obesity, much through its association with insulin resistance [5–7]. However, the AT is not the only source of inflammatory mediators in obesity, since the liver, the pancreas, and the muscle do also contribute to inflammation and are affected by it [3].

In addition, it is well established that the performance of immune cells is largely influenced by metabolic cues and both metabolic and immune systems are highly interdependent [1]. These systems have evolved in close relationship, sharing cellular machinery, being activated by many common hormones, cytokines, signaling proteins, transcription factors, and bioactive lipids and regulating each other. The inflammatory response relies on energetic support from the metabolic system, favoring a catabolic state and suppressing anabolic processes such as those initiated by insulin [8].

Estrogens influence immune and inflammatory processes, as revealed by increased inflammatory responses to infection and sepsis and higher rate of autoimmune diseases in women when compared to men as well as by the variation of chronic inflammatory disease activity with the menstrual cycle, pregnancy, and menopause [9, 10]. Likewise, models of estrogen insufficiency have unveiled new and unexpected roles for estrogens, in both males and females, many of which apply to the regulation of energy homeostasis [11, 12]. However, these two lines of research have been mostly independent and little attention has been drawn towards the possible action of estrogens on the modulation of metabolism through their anti-inflammatory properties. This is a subject of undisputable interest since understanding the regulation of metabolic inflammation by estrogens has the potential of providing new therapies to this increasingly prevalent condition.

In this paper, we will synthesize the current knowledge on how estrogen signaling may modify inflammatory processes thus impacting on metabolism and highlight major research questions on the field.

## 2. Metabolic Inflammation

Inflammation involves the coordinated action of many cell types and mediators in response to harmful stimuli (e.g., physical, chemical, or biological), resulting in neutralization or removal of the insult usually leading to the restoration of homeostasis [13]. It has been highlighted that obesity-associated inflammation does not meet the criteria of classical inflammation, presenting in a peculiar way, not accompanied by infection or signs of autoimmunity, and without extensive tissue injury [1]. Instead, inflammatory activation is often modest and local as compared with that elicited by infection, trauma, or acute immune response [3], being mainly triggered by metabolic surplus [1] with specialized metabolic cells such as adipocytes [3] activating cellular stress pathways [14], thus initiating and sustaining the inflammatory program [3]. It may be understood as a failure to completely restore prior homeostasis, reaching a new equilibrium status (allostasis), with permanently modulated functions [14]. Different proposals have been made to name this inflammatory state as “metaflammation,” meaning metabolically triggered inflammation [1], or “para-inflammation,” defining an intermediate condition between basal and inflammatory states [15]. This has defined the field of immunometabolism as the framework for the occurrence of many metabolic diseases [2, 7]. However, the comprehensiveness of the inflammatory process, as it deals with coagulation, fibrinolysis, complement activation, antioxidation, immune response, and regulation of hormones by the hypothalamic-pituitary-adrenal axis complicates the study of metabolic inflammation. Likewise, in addition to genetic and environmental influences, the possible wide range of mass variability of the tissue most contributing to inflammation, in this case, the AT, makes the subject even more intricate [16].

### 2.1. Initiating Factors and Mechanisms

Under conditions of overfeeding or high fat diets, specialized metabolic cells (e.g., adipocytes, hepatocytes, or myocytes) may engage in cell defensive programs initiating inflammatory activation and communication in response to danger signals. These inflammatory pathways include inflammasome and pattern recognition receptors such as the toll-like receptor (TLR) activation, c-jun N-terminal kinase (JNK), inhibitor of kappaB kinase (IKK)/nuclear factor kappa B (NFkappaB), peroxisome proliferator-activated receptor (PPAR) gamma signaling, and production of inflammatory cytokines [3]. Many of these responses are mediated by disturbances of organelle functioning such as the endoplasmic reticulum or the mitochondria [17, 18]. Affected cells secrete increased amounts of inflammatory cytokines and decreased protective factors (e.g., adiponectin) and the systemic nature of the response is revealed by the increase in circulatory acute-phase reactants (e.g., C-reactive protein, CRP) [7, 19]. Increased release of chemotactic molecules mediates the recruitment of immune cells such as macrophages, mast cells, lymphocytes, eosinophils, and dendritic cells to inflamed tissues [20]. The importance of immune cell infiltration in the AT has been extensively studied and seems to largely contribute to obesity-related metabolic dysfunction and chronic inflammation [21]. Interestingly, over 90% of the cytokines released by AT, with the exception of adiponectin and leptin, can be attributed to cells other than adipocytes [22].

Several possible initiating signals have been proposed for the origin of metabolic inflammation signaling, for example: (i) nutrients may be naturally inflammatory [3]; (ii) increased intestinal permeability after fatty meals or in obese states may allow access of proinflammatory stimuli such as lipopolysaccharide (LPS) to the circulation [23, 24]; (iii) nutrients, not being inflammatory by nature, when in excess initiate immune responses due to a dose-dependent loss of specificity (e.g., excess lipids activating TLRs) [3]. Besides, LPS and nonesterified fatty acids, glucose, hypoxia, cell damage, and death can activate metabolic inflammation in various cells types [6, 8, 25–28]. The repetitive or chronic presence of such stimuli, even of moderate magnitude, either isolated or in combination, disrupts homeostasis creating vicious cycles that reinforce each other [29].

## 3. Estrogens 

Estrogens have well-established roles in reproductive function, but their effects on other systems have been brought to light, namely, in adipose, nervous, cardiovascular, muscle, and bone tissues, implicating these hormones in many diverse physiological roles.

### 3.1. Estrogen Metabolism

**Figure 1:**
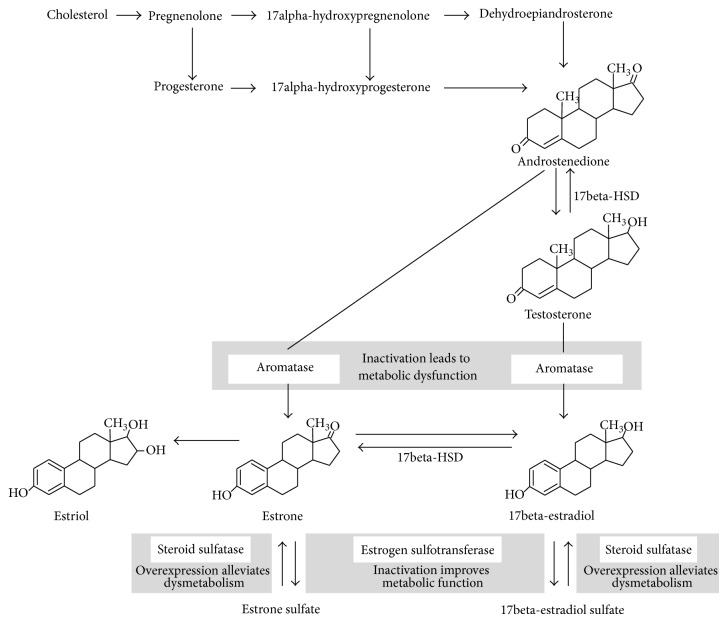
Estrogen metabolism. The effects of experimental manipulation of enzymes marked in grey boxes modulation are highlighted. Inactivation of aromatase leads to metabolic dysfunction that can be reversed by estrogen replacement. In opposite, estrogen sulfotransferase inactivation in models of obesity or type 2 diabetes mellitus improves metabolic function, an effect that is abolished by ovariectomy. Hepatic expression of steroid sulfatase is induced in animal models of obesity and type 2 diabetes mellitus and seems to alleviate dysmetabolic changes; this effect is also lost with ovariectomy. 17Beta-HSD, 17beta-hydroxysteroid dehydrogenase.

Estrogens are synthesized by aromatase, a cytochrome P450 enzyme located in the endoplasmic reticulum of estrogen producing cells which catalyzes the aromatization of testosterone and androstenedione [30], to 17beta-estradiol, the most active estrogen, and estrone, respectively [31]. In humans, estrogens are generated in several tissues including the ovaries, testis, placenta, fetal but not adult liver, bone chondrocytes and osteoblasts, vascular smooth muscle cells, skin, skeletal muscle, several brain regions, and the AT [30, 32, 33]. The gonads and the adrenals express all the necessary enzymes to synthesize estrogens from cholesterol whereas other tissues, such as the bone or the AT, depend on precursor supply from those organs through the blood [34]. In peripheral tissues, 17beta-hydroxysteroid dehydrogenase (17beta-HSD) converts the weaker hormones androstenedione and estrone to stronger ones, testosterone and 17beta-estradiol, respectively (Figure 1) [35, 36].

In premenopausal women, most estrogens are produced in the ovaries, released to the circulation in large amounts and exert their effects in many target tissues through endocrine signaling. After menopause, when estrogen production from the ovaries falls, circulating levels of estrogens decrease dramatically [37] and estrogen synthesis is mainly assured by peripheral sources, primarily by the AT [38]. The AT can contribute with around 100% of circulating estrogens in postmenopausal women [39, 40]. Estrogens are also produced during the entire male life cycle, having an action pattern similar to that of postmenopausal women in which endocrine actions lose relevance to paracrine, autocrine, and intracrine actions in the tissues or in the cells that produce them [30]. Thus, regardless of low circulating levels, local estrogen concentrations may be high, reflecting on their biological actions [41].

Estrogen inactivation occurs via estrogen sulfotransferase which constitutes a critical mediator of hormone action. This cytosolic enzyme conjugates estrogens with sulfate, inactivating them and favoring urinary excretion [12]. Although estrogen sulfates are biologically inactive, they have prolonged half-life in the circulation, acting as a reservoir for regeneration of active estrogens by steroid sulfatase-mediated desulfation [42].

### 3.2. Estrogen Signaling

**Figure 2:**
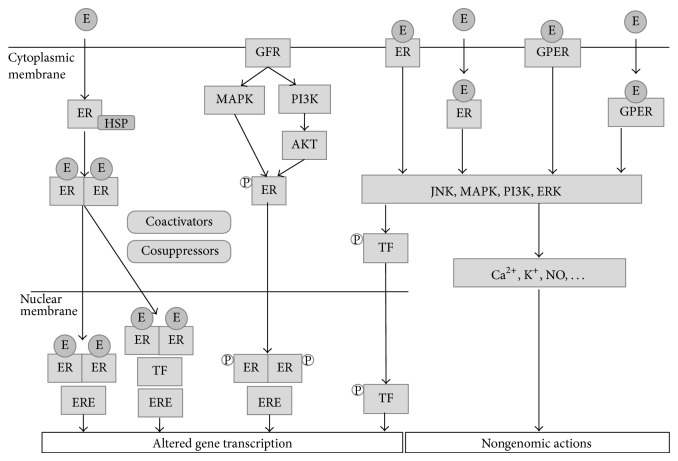
Estrogen signaling occurs through both genomic and nongenomic mechanisms. In classical, genomic, estrogen signaling ERs act as ligand-activated transcription factors, activating or repressing target genes within hours of ligand binding. ERs are located as monomers in the cytoplasm in protein complexes involving heat-shock proteins and estrogen binding promotes their dissociation from this complex and ER dimerization. ER dimers bind directly to estrogen response elements of target gene promoters, or indirectly through interaction with other DNA-bound transcription factors. ERs also regulate gene expression in a ligand independent manner being activated downstream to growth factors binding to growth factor receptors, through the action of intracellular kinases or though the formation of heterodimers with different nuclear receptors (not shown). Genomic actions are modulated by cell-specific interaction with cofactors (coactivators or cosuppressors). Metabolic effects of estrogens seem to be largely mediated through nonnuclear ERs, either by interference with gene expression or by exerting nongenomic actions. This involves activation of ERs and G-protein-coupled ER located at the membrane or at extranuclear sites within seconds or minutes resulting in changes in Ca^2+^, K^+^, cAMP, and nitric oxide levels, activation of G protein-mediated events, and stimulation of different types of kinases such as extracellular-regulated kinases, phosphoinositide 3-kinases, mitogen-activated protein kinase, and c-Jun N-terminal kinases. E: estrogen; ER: estrogen receptor; ERE: estrogen-responsive element; ERK: extracellular-regulated kinase; GFR: growth factor receptor; GPER: G protein-coupled estrogen receptor; HSP: heat-shock protein; JNK: c-Jun N-terminal kinase; MAPK: mitogen-activated protein kinase; NO: nitric oxide; PI3K: phosphoinositide-3 kinase; TF: transcription factor.

Estrogen signaling occurs through both genomic and nongenomic mechanisms summarized in Figure 2. Classical, genomic, estrogen signaling has been described to occur through specific nuclear receptors acting as ligand-activated transcription factors [12, 43], the estrogen receptors (ERs), of which two isomers are known: ERalpha and ERbeta [9, 44, 45]. ER distribution varies among tissues resulting in distinct effects of the hormone in different locations and the relative amount of each subtype may also change estrogen effects [9]. ERalpha is the predominant receptor in the kidney [43], heart [46], bone, uterus, liver, and AT [47], whereas ERbeta is the predominant receptor in the ovary, prostate, lung, bladder, hematopoietic cells, gastrointestinal tract, and central nervous system [43, 47].

Classical genomic signaling through the ERs occurs within hours of ligand binding, activating or repressing target genes. ERs are located as monomers in the cytoplasm in protein complexes involving chaperone heat-shock proteins and estrogen binding promotes their dissociation from this complex and ER dimerization (homodimers of ERalpha or ERbeta or heterodimers of ERalpha-ERbeta). ER dimers bind directly to estrogen response elements of target gene promoters, or indirectly through interaction with other DNA-bound transcription factors like activator protein 1 or specific protein 1. ERs also regulate gene expression in a ligand independent manner by interacting with other nuclear hormone receptors, such as PPARs [48]. After DNA binding, ER dimers regulate gene expression interacting with cofactors (coactivators or cosuppressors) [12, 43, 46]. The nature and concentration of the ligand, the type of dimer formed, the kind of DNA interactions (direct or indirect), and the presence of distinct cofactors according to cell type or condition all constitute sources of variation of ER-activated transcriptional activity. Furthermore, there appears to be ERalpha dominance since when both receptors are present, this receptor seems to be the driver of the response for either genomic or nongenomic responses, while ERbeta when in the presence of ERalpha tends to antagonize its responses [49].

While most of the reproductive effects of estrogens are mediated through classical ER signaling, metabolic effects seem to be largely mediated through nonnuclear ERs, either by interference with gene expression or by exerting nongenomic actions. This involves activation of ERs located at the membrane or at extranuclear sites within seconds or minutes [12, 50]. Mechanisms of nongenomic actions are not completely known but second messenger activation after estrogen binding results in changes in Ca^2+^, K^+^, cAMP, and nitric oxide levels, activation of G protein-mediated events, and stimulation of different types of kinases such as extracellular-regulated kinases (Ras/Raf/MEK/ERK), phosphoinositide 3-kinases (PI3K), p38 mitogen-activated protein kinase (MAPK), and JNK [51–53]. MEK/ERK pathway activation also regulates gene expression through activation of transcriptional factors such as cAMP-related element binding (CREB) protein or nuclear factor of activated T-cells [54].

G protein-coupled receptor 30, now known as G-protein-coupled ER (GPER), located on the plasma membrane [55] or with an intracellular localization [56] is also involved in rapid nongenomic estrogen signaling involving release of intracellular Ca^2+^ and activation of calcium-calmodulin-dependent kinases, PI3K and MAPKs [43].

Estrogen-related receptors (ERRs), a family of orphan receptors closely related to ERs, include three members, ERRalpha, ERRbeta, and ERRgamma, and are expressed in muscle, heart, bone, and AT [57]. These nuclear receptors are not stimulated by estrogens or estrogen-like molecules, being constitutively active in the absence of ligand [58]. However, ERRs can interfere with estrogen signaling, recognizing the same DNA-binding elements as ERs and sharing common target genes [57]. ER and ERR are coexpressed in many tissues and ERRs play important functions in adaptative energy metabolism [58].

With much relevance to the inflammation modulatory properties of estrogens is their capacity to influence NFkappaB activity through genomic and nongenomic mechanisms. NFkappaB is a central regulator of a variety of proinflammatory genes. It represents a homo- or heterodimeric complex of the REl family of proteins comprising p65 (Rel A), p50/105, p52/100, and Rel B. In its inactive form in unstimulated cells, NFkappaB resides in the cytoplasm bound to IkappaB. Upon stimulation achieved by different stimuli such as oxidative stress, viruses, and cytokines, IkappaB is phosphorylated mainly by IKK leading to IkappaB/NFkappaB complex dissociation and IkappaB ubiquitination followed by proteasome mediated degradation. This allows formation of NFkappaB dimers (heterodimer formation is necessary for transcriptional activation) that translocate to the nucleus, bind to kappaB sites on target genes, and modulate expression of genes involved on inflammatory pathways and protective cellular responses. However, sustained NFkappaB activation can lead to chronic inflammation [46, 59, 60].

Estrogens may interfere with KFkappaB signaling by multiple mechanisms. ERs can bind to c-Rel and RelA in a ligand-independent manner interfering with the formation of active NFkappaB dimers [61–63]. Additionally, the atherogenic diet-induced* in vivo* hepatic NFkappaB activation was blocked by estrogen replacement in an ER-dependent manner [64]. Estrogens also indirectly inhibit NFkappaB DNA binding, as they have been shown to inhibit IKK activation, increase IkappaB expression, and decrease its phosphorylation [60, 65–68]. Furthermore 17beta-estradiol, membrane impermeable 17beta-estradiol, and overexpression of ERalpha may inhibit inflammatory activation mediated by NFkappaB and JNK via PI3K/AKT [69]. However, effects obtained vary according to cell type, relative abundance of the different ER subtypes, and timing at which effects are observed following estrogen exposure [46].

## 4. Estrogen Signaling in Metabolic Inflammation

Given the close interplay between both processes, it is impossible to approach the effects of estrogens on inflammation without having in mind that they exert a profound impact on metabolic regulation, the reverse also being true. However, in the past, most research has dealt with these two matters independently and only recently the modulation of metabolic dysfunction through changes in inflammation by estrogens is beginning to be unraveled. Even so, this kind of association has been more frequently established in regard to cancer, osteoporosis, cardiovascular disease, neurodegeneration, and stroke [70–74], still leaving many questions blank concerning obesity and insulin resistance. AT dysfunction has been considered a key initiating factor and the main source of inflammatory signaling in obesity-associated metabolic alterations, along with the resulting insulin resistant state [17, 75]. These two processes will therefore be the main scope of this review regarding their modulation by estrogen signaling, underscoring inflammatory mediation.

### 4.1. Energy Balance

Estrogens influence energy balance being usually regarded as opposing excessive body fat accumulation [76]. Multiple mechanisms account for this activity including actions on the central nervous system to regulate food intake and energy expenditure [77, 78] and direct effects on the physiology of the main energy metabolism-regulating organs such as the AT, the muscle, the liver, and the pancreas, where they regulate lipid and glucose metabolism, and also on immune cells [11, 12].

Estrogens decrease food intake and favor energy expenditure [78]. Ovariectomy leads to increased adiposity, partially by increasing food intake, which is prevented by estrogen treatment. In the same line, aromatase inactivation results in obesity without increasing food intake. Instead, aromatase knockout mice from both sexes show a decrease in spontaneous physical activity and decreased lean mass. ERalpha deficiency also increases food intake and decreases energy expenditure revealing the involvement of this ER subtype in energy balance regulation [12, 50].

### 4.2. Estrogens and Leptin

The regulation of leptin signaling by estrogens has been documented and may contribute to estrogens' effects on energy balance. Leptin receptor on the hypothalamus colocalizes with ERalpha and estrogen treatment decreases the expression of leptin receptor on the arcuate nucleus, where leptin exerts its catabolic actions being anorexigenic and increasing energy expenditure. Leptin is produced by adipocytes and the subcutaneous AT contributes most to leptin secretion due to higher mass (subcutaneous fat is the major fat storage depot in men and women) and higher expression in this location [79]. Leptin expression is highly influenced by estrogens [80], which are also produced in higher amounts in subcutaneous AT [81]. In addition, estrogen treatment has been shown to increase leptin sensitivity in the brain both in males and in females while ovariectomy in females reduces it. Multiple factors, including inflammation and endoplasmic reticulum stress, have been shown to induce leptin resistance [82]. Furthermore, apart from its metabolic roles, leptin is an important mediator of immune responses and inflammation. Besides having direct roles in immune cells and inflammatory reactions [83, 84], leptin also influences inflammatory processes by mediating trade-offs between the immune system and other physiological systems to balance allocation of energy to inflammatory processes [85]. Therefore, the interaction between estrogens and leptin constitutes a possible indirect link between metabolic and inflammatory pathways.

### 4.3. Estrogens and Glucocorticoids

Estrogens' influence on the hypothalamus-pituitary-adrenal-axis is another interesting hypothesis on how estrogens may impact on metabolic inflammation. Excess glucocorticoid signaling has been implicated in the development of visceral obesity and insulin resistance [86]. It has been proposed that glucocorticoid levels are elevated to counter inflammation in obesity and metabolic dysfunction resulting in insulin resistance, altered lipid metabolism, favored fat deposition, and protein mobilization. Estrogens may limit these actions of glucocorticoids and the inflammatory stimuli that lead to their elevation and fluctuations in estrogen levels during the life cycle can determine susceptibility to metabolic disorders [87, 88].

Estrogens are able to regulate the hypothalamus-pituitary-adrenal-axis through intrahypothalamic effects in the basal state and during restraint stress, an effect mediated by ERalpha and by both ERalpha and -beta, respectively [89]. Furthermore, the oleoyl ester of estrone, the storage from the AT of estrone, the main estrogen synthesized in this tissue from circulating androstenedione, regulates adrenal synthesis of corticosteroids. Oleoyl-estrone increased adrenal tissue corticosterone levels and gene expression of glucocorticoid-synthesizing enzymes reflecting higher serum corticosterone, while also increasing liver expression of corticosteroid-disposing steroid 5alpha-reductase, suggesting an increase in glucocorticoid turnover [90]. However, estrogens may also act by regulating glucocorticoid metabolism in peripheral tissues due to their ability to modulate 11beta-hydroxysteroid dehydrogenase type 1 (11beta-HSD1), the enzyme that activates circulating cortisone to cortisol (or 11-dehydrocorticosterone to corticosterone in rats), regulating glucocorticoid availability in a tissue-specific manner at the prereceptor level [91]. This enzyme modulates endogenous glucocorticoid action: acute inflammation is more evident in 11beta-HSD1 deficiency or inhibition. However, in obesity or diabetes, two chronic inflammatory conditions, reduced 11beta-HSD1 activity may be beneficial, contributing to decreased inflammation [92]. Estradiol treatment to ovariectomized rats downregulated liver and visceral, but not subcutaneous, AT 11beta-HSD1 activity and expression. This resulted in an alteration of the ratio of 11beta-HSD1 expression and activity between subcutaneous and visceral AT where estradiol-treated rats displayed increased subcutaneous compared to visceral 11betaHSD1, contrary to what was observed in untreated ovariectomized rats [93]. Using 3T3-L1 cells and adipocytes isolated from mesenteric fat depots, it has been shown that 17beta-estradiol treatment reduces 11beta-HSD1 expression within 5–10 minutes of stimulation without interference of ERs [94].

These actions are very relevant because, by decreasing glucocorticoid availability, estrogens oppose to their effects on the promotion of metabolic disturbances. On the liver, chronic 17beta-estradiol administration resulted in substantial inhibition of 11betaHSD1 mRNA expression and enzyme activity. Inhibition of 11beta-HSD accounted for reduced phosphoenolpyruvate carboxykinase, the rate-limiting step in gluconeogenesis [95]. On adipocytes, glucocorticoids potentiate adipogenesis and anabolic lipid metabolism and estrogens are described to have opposite effects [94]. Expression of 11beta-HSD1 in human adipocytes is induced by proinflammatory cytokines such as interleukin (IL) 1beta and tumor necrosis factor (TNF) alpha through signaling mechanisms including CCAAT/enhancer binding protein beta, MAPK/ERK kinase, and NFkappaB/RelA [96] and at least the latter two are also estrogen targets [46, 55], constituting a possible site of regulation.

### 4.4. Adipose Tissue Distribution

Estrogens regulate not only the amount of body fat accumulated but also its distribution among different depots. Men have lower amount of total body fat than women that, while in the premenopausal period, have a greater proportion of body fat accumulated in the subcutaneous (gluteofemoral) location than men, although in both genders the subcutaneous is the main site of accumulation [12, 79]. Thus men accumulate more fat in the visceral compartment compared to premenopausal women. However, after menopause, body fat is redistributed and women tend to acquire a body fat accumulation pattern similar to men [12, 50] and this redistribution may be linked to the age-related decrease in estrogen levels [93].

Premenopausal women are less prone to develop cardiovascular and metabolic diseases than age-matched men, illustrating estrogen protective effects [97, 98]. Men and postmenopausal women, whose estrogen levels are low, have increased metabolic risk translated into a higher likelihood to develop cardiovascular diseases, type 2 diabetes, and certain forms of cancer [99]. The increase in cardiometabolic risk after menopause is paralleled by the redistribution of body fat and is reversed or decreased by hormone replacement therapy (HRT) [50, 88, 100]. Frequently, several altered metabolic markers are clustered in the same individual justifying the increased risk. Probably one of the most prevalent alteration is visceral or central obesity, the main component of the metabolic syndrome, which also includes insulin resistance, low high density lipoprotein levels (HDL), hypertriglyceridemia, and hypertension [101].

AT pathogenicity markedly differs according to location of AT deposition (visceral or subcutaneous) [102]. Classical AT functions include heat insulation, mechanical cushioning, and storage of triacylglycerols [103]. Presently, it is known that the AT secretes endocrine, paracrine, and autocrine active substances in response to different stimuli [79, 104]. Some are specific to adipocytes, such as the adipokines leptin and adiponectin, and other may be produced by several cell types in the AT and include inflammatory cytokines (e.g., TNFalpha, IL6), chemokines (monocyte chemoattractant protein 1, MCP1), acute phase reactants, components of the alternative complement system, eicosanoids, and molecules with anti-inflammatory properties [105].

Visceral fat is a highly metabolic tissue. It is apparently more susceptible to lipolysis than subcutaneous AT [106] presenting denser irrigation and innervation than subcutaneous AT and draining secretions into the hepatic portal vein [107]. Excess accumulation of AT in the visceral compartment seems to independently predict insulin sensitivity [108–110], impaired glucose tolerance [111], elevated blood pressure [112, 113], and dyslipidemia [109, 114]. Regarding proinflammatory cytokines, visceral AT is associated with higher production of TNFalpha [106, 115], plasminogen activator inhibitor 1 (PAI1) [116], IL6, and CRP [117], while producing lower amounts of the anti-inflammatory, insulin-sensitizing adipokine adiponectin, which correlates more strongly with subcutaneous fat [118]. Currently, there is the suggestion that subcutaneous fat depots are protective [50, 88] and that the lack of AT, as evident in lipodystrophy [119], may equally lead to the development of metabolic syndrome.

Adipocytes from different depots differ in their capacity to produce estrogens, as aromatase expression declines from gluteofemoral, to subcutaneous abdominal, to visceral AT [120, 121]. Estrogens are secreted from AT in proportion to total fat mass [122] and are thought to signal the size of body energy stores and influence physiological processes through endocrine actions while also exerting important local effects through paracrine and autocrine actions.

The absence of estrogen production in the ovaries after menopause by increasing adiposity may augment AT synthesis of estrogens. It is known thatobese postmenopausal women have higher serum estrogen concentration than lean postmenopausal women [123] and the proinflammatory condition associated with obesity may play a part in this association since aromatase is induced by cytokines such as TNFalpha [124]. Given 17beta-estradiol's anti-inflammatory properties, for example, through suppression of IL6 or TNFalpha secretion by macrophages and dendritic cells [9], estrogens may have a paracrine role in AT inflammation targeted to alleviate potential tissue-damaging effects.

### 4.5. Changes in Estrogen Signaling and Availability

**Figure 3:**
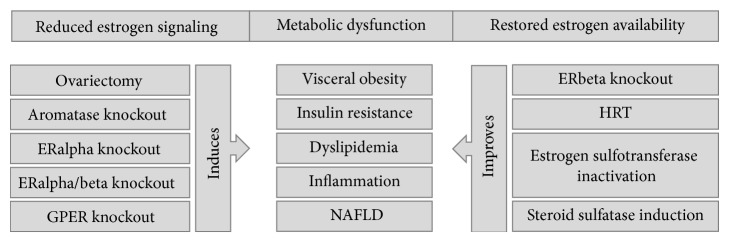
Different models have shown the influence of estrogens on metabolic-related inflammation. Loss of/decreased estrogen signaling through decreased production of estrogens or ERalpha, ERalpha/beta, or GPER inactivation promotes metabolic dysfunction revealed by visceral obesity, insulin resistance, dyslipidemia, inflammatory activation, and nonalcoholic fatty liver disease. On the other hand, promoting maintenance of estrogen signaling through hormone replacement therapy, blocking estrogen inactivation by estrogen sulfotransferase or increasing its reactivation from the estrogen-sulfate circulating pool by steroid sulfatase induction, tends to counteract metabolic dysfunction. Interestingly, inactivation of ERbeta also promotes metabolic health, showing the opposite metabolic effects mediated by both ER receptors. ER: estrogen receptor; GPER: G protein-coupled estrogen receptor; HRT: hormone replacement therapy; NAFLD: nonalcoholic fatty liver disease.

The absence of estrogens in animal and human models appears to favor the occurrence of the metabolic syndrome, starting with the facilitation of visceral AT deposition, which is reversed or prevented by estrogen reposition (Figure 3) [125].

*Ovariectomy*. Ovariectomy in laboratory animals results in the increase of AT accumulation, mainly visceral AT. Estrogen treatment in ovariectomized rats reduces adipocyte size by reducing fatty acid uptake, through a downregulation of lipoprotein lipase, and lipogenesis, downregulation of acetyl coenzyme A carboxylase, and fatty acid synthase [126]. Furthermore, the increase in fat mass is accompanied by a decrease in insulin sensitivity and glucose intolerance while circulating low density lipoprotein (LDL), triglycerides, fatty acids, and proinflammatory markers become elevated [127]. 17Beta-estradiol treatment reverses the impaired glucose tolerance, hyperglycemia, and reduced insulin release caused by ovariectomy in rats [128]. The protective effects of chronic 17beta-estradiol replacement on glucose intolerance are less obvious after feeding with normal diet than with high-fat diet in ovariectomized rats. However, 17beta-estradiol increased the transcription of IL6, TNFalpha, and plasminogen activator inhibitor 1 (PAI1) induced by high-fat diet in the liver and visceral AT, while maintaining the beneficial effect on glucose tolerance. These apparently contradictory results were interpreted as a dissociation between the metabolic and inflammatory effects of estrogens [129].  Table 1 summarizes the main effects of estrogens with relevance to metabolic inflammation.

*Aromatase*. Men with mutations leading to aromatase inactivation have central obesity and elevated plasma LDL cholesterol and triglycerides and insulin resistance revealed by high insulin in the presence of normal glucose levels [130]. Male and female aromatase knockout mice also present metabolic dysfunction characterized by intraabdominal obesity, insulin resistance, dyslipidemia, and hepatic steatosis [78, 125, 131]. Hepatic steatosis was associated with impairment in fatty acid beta-oxidation, alterations that were reversed by administration of 17beta-estradiol [132].

*Estrogen Receptors*. ERalpha signaling mediates many important metabolic effects of estrogens both in males and in females. Several studies have shown associations between ERalpha polymorphisms and increased AT accumulation, particularly in the visceral compartment [133, 134]. In rodents, the inactivation of ERalpha is, in many aspects, similar to the loss of the aromatase gene. ERalpha or double ERalpha and -beta knockouts also show increased intraabdominal AT, adipocyte hypertrophy, insulin resistance, and decreased glucose tolerance independently of gender [135]. In addition, ERalpha knockouts have reduced oxygen uptake and caloric expenditure and present increased fasting insulin, leptin, and PAI1 levels while adiponectin is reduced under normal diet. Along with impaired glucose tolerance and skeletal muscle insulin resistance that are enhanced under high fat feeding, accumulation of bioactive lipid intermediates, inflammation, and diminished PPARalpha, PPARdelta, and uncoupling protein (UCP) 2 transcript levels are also evident [136]. Interestingly, ovariectomized ERalpha knockouts present a normalized homeostasis of circulating glucose and insulin levels and a reversal of the obese phenotype, proposing that ERbeta-associated estrogen action contributes to metabolic dysfunction. Indeed, ERbeta inactivation in mice leads to improved insulin sensitivity and glucose tolerance without increasing body fat content which supports the occurrence of opposite metabolic effects of estrogens on each of these receptors [137]. However, both ERalpha and ERbeta interfere with AT distribution, inflammation, fibrosis, and glucose homeostasis. Selective deletion of AT ERalpha in adult mice reproduces the findings from total body inactivation in ERalpha. More recently, a mouse model with ERalpha inactivation specifically in adipocytes (adipoERalpha) was generated. These mice displayed increased AT fibrosis and inflammation which was more marked in males, showing that the protective effects of estrogens on AT are dependent upon adipocyte ERalpha-mediated signaling [138].

*G Protein-Coupled Estrogen Receptor*. A role for GPER-mediated estrogen signaling in controlling energy homeostasis and development of obesity has been established [139]. GPER-deficient mice are also obese, supporting a role for GPER-dependent estrogen signaling in counteracting obesity development in both genders [140]. By 6 months of age GPER knockouts of both genders were obese, without changes in food intake or locomotor activity, and insulin resistant but only females exhibited glucose intolerance. At one year of age males had higher cholesterol, triglyceride levels and by two years of age had increased IL1beta, IL6, IL12, TNFalpha, MCP1, interferon gamma-induced protein 10, and monokine induced by interferon gamma and decreased adiponectin circulatory levels than wild type littermates [141]. GPER also modulates estrogen-related insulin release and beta-cell survival. GPER1 knockouts have impaired glucose tolerance and decreased glucose-stimulated insulin secretion [142] and 17beta-estradiol-stimulated insulin release is abolished in GPER1 knockout mice. Furthermore, part of the protective effects of estradiol on beta-cell death induced by oxidative stress are mediated through GPER estrogen signaling [143].

*Estrogen-Related Receptors*. ERRalpha regulates mitochondrial function and metabolism and has also been shown to mediate bone-derived macrophage activation by proinflammatory cytokines [144]. ERRalpha null mice are lean and resistant to high fat diet-induced obesity. In these mice, UCP1 expression is upregulated in white AT possibly contributing to increased energy expenditure and lower body weight [57]. Acute-phase response induced by LPS, zymosan, or turpentine in mice reduced mRNA levels of ERRalpha, its coactivator, PPAR gamma coactivator 1 (PGC1) alpha, and medium-chain acyl-coenzyme A dehydrogenase, one of its targets, in the liver, heart, and kidney. This regulation was associated with the decrease in fatty acid oxidation and hypertriglyceridemia during inflammation and infection [145]. ERRgamma is another constitutively active transcription factor regulating genes involved in hepatic glucose metabolism and the endoplasmic reticulum stress. An interesting link between ERRgamma transcriptional activity and metabolic inflammation has been established by the demonstration that overexpression of this receptor led to regulation of cAMP responsive element-binding protein H (CREBH), an endoplasmic reticulum-bound transcription factor that mediates endoplasmic reticulum stress by increasing expression of acute-phase proteins in response to inflammation. Upon ERRgamma activation both CREBH and CRP expression were upregulated in a PGC1alpha-dependent manner, while ERRgamma knockdown or inhibition reduced these effects. Moreover, ERRgamma inhibition was also able to reduce augmented CRP gene expression in a model of obese diabetic animals [146].

*Estrogen Sulfotransferase and Steroid Sulfatase*. Estrogen sulfotransferase has been shown to have an important role in energy balance and type 2 diabetes pathogenesis [12]. Inactivation of estrogen sulfotransferase in female mice improves metabolic function in ob/ob, dexamethasone-, and high-fat diet-induced mouse models of type 2 diabetes. These mice display improvements of body composition, higher energy expenditure, and insulin sensitivity and decreased hepatic gluconeogenesis and lipogenesis compared to controls. These effects are abolished by ovariectomy and seem to result from increased estrogen availability and action on the liver. Metabolic improvement was only evident in females, whereas ob/ob males displayed worse metabolic prolife than controls [147]. Estrogen sulfotransferase is present in abdominal subcutaneous AT of both obese males and females being positively correlated with mRNA levels TNFalpha and suppressor of cytokine signaling 3, suggesting its association with inflammation [148]. Corroborating this assumption, functional depletion of estrogen sulfotransferase in the human umbilical vein endothelial cell line resulted in regulation of genes involved in inflammation and lipid metabolism. Anti-inflammatory cytokines (IL4 and IL10) were upregulated and proinflammatory cytokines were downregulated (TNFalpha, IL1beta, and IL6, among others) and genes involved in cholesterol uptake and cholesterol ester synthesis were also increased. This regulation was attenuated by PPARgamma knockdown or antagonism, meaning that sulfotransferase activity upregulates PPARgamma in the presence of 17beta-estradiol [149]. Steroid sulfatase-mediated desulfation is another mechanism that modifies estrogen availability, regulating its actions. In genetic (ob/ob) or environmental (high-fat diet-induced) models of obesity or of type 2 diabetes steroid sulfatase was induced in the liver. This increase of steroid sulfatase in the liver of transgenic mice has been interpreted as a mechanism to counterbalance metabolic dysfunction, since overexpression alleviated dysmetabolism in high-fat diet and ob/ob models of obesity and type 2 diabetes, resulting in reduced body weight, improved insulin sensitivity, and decreased hepatic steatosis and inflammation. This protection in females was proposed to be mediated through reactivation of circulating estrogen sulfates to active estrogens what was supported by the observation that metabolic amelioration induced by steroid sulfatase overexpression was lost in ovariectomized rats [150].

*Hormone Replacement Therapy*. It is generally accepted that a great part of the increase in cardiometabolic risk after menopause is accounted for by the decline in ovarian production of estrogens and accumulating evidence argues in favor of risk reduction with HRT [151]. Despite the report from Women's Health Initiative of increased incidence of cardiovascular events in postmenopausal women with ages between 50 and 79 receiving HRT [152] having led to the early termination of this large-scale trial, further analysis of study data, separating cohorts by age, revealed a protection in women with fewer menopausal years (50 to 59 years of age) [153]. Presently, extensive research supports that HRT has an important impact on both metabolic and inflammatory processes [151]. In postmenopausal women, estrogen therapy decreased the expression of genes involved in lipogenesis including acetyl-coenzyme A carboxylase, sterol regulatory element-binding protein 1c, stearoyl-CoA desaturase, lipoprotein lipase, fatty acid synthase, fatty acid desaturase, and PPARgamma in human abdominal AT [154, 155]. Conjugated estrogens and estradiol administered to postmenopausal women led to an increase in glucose disposal during hyperinsulinemic-euglycemic clamp studies [156, 157]. Human studies have also reported increased circulatory cytokine levels after menopause, including TNFalpha, IL1, and IL6, these levels being substantially lower in women receiving HRT [158]. In parallel, plasma levels of MCP1 have been reported to be lower in postmenopausal women receiving HRT compared to those not receiving HRT [159]. The observation that HRT results in increased CRP led to the suggestion that it induces inflammation that may be responsible for acute cardiovascular events. However, by evaluating vascular inflammation markers in postmenopausal women with increased cardiovascular risk after HRT or no HRT, it was demonstrated that although CRP levels did increase after HRT, all other inflammation markers were decreased (soluble intracellular adhesion molecule 1, vascular cell adhesion molecule 1, E-selectin, and IL6), while no significant changes were observed in women not taking HRT. The authors concluded that metabolic hepatic activation is responsible for increased CRP instead of an acute-phase response and that HRT overall decreases vascular inflammation [160]. Another important addition to this debate is the fact that a formulation that includes a progestin could exert a dose-dependent inhibitory effect on circulating adiponectin and that women with central obesity, which is also associated with the highest levels of free estrogens, or with insulin resistance may not benefit from estrogen progestin therapy [161]. Furthermore, the effect of HRT upon plasma inflammation markers may be dependent upon route of administration, since oral therapy has been shown to be associated with better inflammatory profile than transdermal therapy [162].

### 4.6. Estrogens and Adipose Tissue Cellularity

Obesity is described to be the result of an increase in the number and in the size of adipocytes [163]. Indeed, these are defining features of obesity as this dynamic organ expands to store excess energy. Consequently, for some time it was belived that decreasing both adipose hyperplasia and hypertrophy were necessary measures to overcome obesity and its complications. However that concept is no longer defensible since it was demonstrated that AT expandability is needed to buffer nutrient excess and only when this capacity becomes exceeded other disturbances arise [28, 164–167]. In this regard, an “unorthodox therapeutic strategy” for the treatment of obesity-associated metabolic complications has been proposed: to increase adipocyte recruitment and facilitate the development of hyperplastic forms of AT [168].

Indeed, hypertrophic AT growth seems to associate with adipocyte dysfunction resulting in obesity-related metabolic disorders. In hypertrophic cells, both mechanic and hypoxic stresses are increased inducing the production and release of proinflammatory molecules and predisposing cells to apoptotic death or necrotic cell rupture [28, 169]. This has been considered one of the most plausible causes of inflammatory cell recruitment to AT of obese animals where macrophages surround dead adipocytes [170]. For each dead adipocyte, several macrophages are recruited [171], implying a large amplification of the inflammatory response since macrophages are thought to locally produce inflammatory mediators. Moreover, it has been proposed that visceral AT growth is mainly due to hypertrophy, while in other locations there may be mainly growth through hyperplasia [172]. Illustrating hypertrophic AT pathogenicity, bigger adipocytes are found in insulin resistant patients [173] and adipocyte size is positively correlated with plasma levels of TNFalpha, IL6, and high sensitivity CRP levels and negatively correlated with adiponectin [174]. Furthermore, the insulin-sensitizing anti-inflammatory thiazolidinediones have a proadipogenic effect, resulting in a higher capacity of fat storage by the AT [168]. Curiously, it has been shown that macrophage infiltration on the AT is markedly reduced after surgery-induced weight loss in morbidly obese individuals, the remaining macrophages expressing anti-inflammatory cytokines like IL10 [175].

The determination of adipocyte size depends on the balance between the lipogenic and lipolytic pathways but also on the rate of adipocyte proliferation and differentiation and estrogens influence all these processes [76]. The stromal fraction of AT is composed of several cell types, including preadipocytes and endothelial and vascular smooth muscle cells, most of which respond to estrogens in the surrounding environment, some being able to produce them [76, 176].

Estrogens can directly inhibit AT deposition by decreasing lipogenesis. This effect results mainly from decreasing activity of lipoprotein lipase. Ovariectomy increases lipoprotein lipase and lipid deposition in adipocytes and 17beta-estradiol reverses this increase [177]. On the other hand, 17beta-estradiol can indirectly affect lipolysis by inducing the lipolytic enzyme hormone-sensitive lipase [178] or by increasing the lipolytic effects of adrenaline [179]. Moreover fatty acid oxidation might also be increased, which might contribute to the decrease in AT mass induced by 17beta-estradiol. However, contrary to its antilipogenic and lipolytic effect, estrogen attenuates the lipolytic effects of adrenaline on the subcutaneous AT by increasing the expression of alpha2aA-adrenergic receptors the subcutaneous AT [79]. This effect could in part account for the increase deposition of subcutaneous AT in women compared to men [180]. Estrogen effects in the inhibition of lipogenesis and triglyceride accumulation are also evident after high-fat diet feeding [181].

In the AT, the highest aromatase expression is found in preadipocytes although vascular cells also present aromatase activity [176]. As preadipocytes undergo differentiation, aromatase expression decreases being almost undetectable in fully differentiated adipocytes [30]. Thus the higher estrogen production by the subcutaneous AT may constitute a marker of functional AT that retains its capacity to buffer nutrient excess and avoid visceral obesity and ectopic fat accumulation.

Estrogen signaling through inositol-3-phosphate pathway is involved in preadipocyte proliferation and differentiation into mature adipocytes [182]. These effects are likely to involve ERalpha, protein kinase C, and MAPK. Despite this description, estrogen effects on proliferation are still not very clear. These hormones have been shown to decrease preadipocyte number, particularly by limiting the exaggerated rate of cell divisions that occurs in their absence [76]. On the other hand, during puberty, women acquire more AT, characterized by hyperplasia [183]. Increased overall AT mass in women partially reflects a greater number of adipocytes compared to men pointing towards a role of estrogens in adipocyte development and establishment of adult adipocyte number, as well as in the modulation of adipocyte size in adult females. Similarly 17beta-estradiol stimulates proliferation of subcutaneous rat preadipocytes from females, but not males, and the same phenomenon occurs in human-derived cells [184]. Accordingly, 17beta-estradiol may act as an important local factor influencing the proliferation of preadipocytes that may affect adipocyte number in a depot- and gender-specific manner in human abdominal subcutaneous and omental AT [185]. Moreover, both male and female ERalpha knockouts have large increases in fat pad weight, which results from hyperplasia more than hypertrophy of adipocytes [186]. Similar results were seen in aromatase knockout mice [78], in which the lack of estrogen signaling led to a large increase in the number of adipocytes, demonstrating that estrogen normally plays an inhibitory role during adipogenesis to limit adipocyte number.

Concerning differentiation, results also point to different directions. There is evidence that estrogens are proadipogenic hormones while some studies show that estrogens inhibit estrogen differentiation [76]. However, despite being considered to facilitate preadipocyte differentiation, they are usually related to a decrease in total body fat and in adipocyte hypertrophy [76]. In nonovariectomized female and ovariectomized female mice supplemented with estrogen there is a strong inhibition of key adipogenic genes. However, male mice and ovariectomized females gain weight predominately in the form of abdominal AT possibly due to an increase in adipocyte size [187]. Notwithstanding, it is possible that the reported decrease in adipogenic genes reflects the hyperplastic, rather than hypertrophic nature of AT under the influence of estrogens, a shift that may underlie many of the metabolic and inflammatory benefits of these hormones.

### 4.7. Estrogens and Inflammatory Cells

The increase in the number of AT macrophages in obesity has been set into attention with the work of Weisberg et al. [188]. Several differences have been encountered between sites of AT deposition both in the type and number of inflammatory cells that they may host and in the production and integration of metabolic and inflammatory signals. Both in humans and in rodents, adipocyte size is a strong and direct predictor of macrophage accumulation in the AT [189, 190]. When the diameter of adipocytes increases and the ability of adipocytes to expand reaches a limit AND fail accommodate the excess of nutrients, cells become a source of adipokines such as TNFalpha, IL6, and MCP1, consolidating the perpetuation of inflammation. Macrophage infiltration was found to be higher in visceral compared with subcutaneous fat pads in a mouse model of obesity and correlated with adipocyte size [188, 191]. However, omental adipocytes exhibit smaller adipose cell size than subcutaneous ones, showing that adipocyte hypertrophy is not the sole determinant of macrophage recruitment to the AT, highlighting the importance of factors related AT location [189, 190]. It has also been shown that most of the inflammatory cytokine production from the AT of obese individuals is derived from inflammatory rather than from adipose cells [1]. This suggests that these cells have paramount importance in the genesis of obesity complications and infiltration of the AT by macrophages constitutes also an important point of possible regulation by estrogens.

ERalpha and ERbeta are present in monocytes and macrophages, and estrogens activate these cells [70, 192]. Apparently, peripheral blood mononuclear cells (PBMCs) with exception of monocytes of postmenopausal women have similar ER expression patterns compared to those of premenopausal females. ERalpha is higher in monocytes from postmenopausal women which may result in altered estrogen responsiveness compared with monocytes from premenopausal women [193]. However, results on the effect of estrogens on PBMCs are also conflicting. After menopause, IL6 expression may be present in nonstimulated PBMC isolated directly from venous blood, which suggests the possibility of an endogenous activation of these cells* in vivo*, possibly due to the absence of circulating estrogens [194]. In the presence of LPS, 17beta-estradiol inhibited TNFalpha secretion from PBMCs at concentrations of 10^−10^ to 10^−7 ^mol L^−1^ in male subjects and at 10^−8^ to 10^−7^ mol L^−1^ in female subjects but had a stimulating effect in the absence of LPS [195]. In human whole blood cultures, 17beta-estradiol at 10^−10^ to 10^−8^ mol L^−1^ decreased spontaneous secretion of IL6, TNFalpha, IL1 receptor antagonist, IL1beta, and the ratio of IL1beta/IL1 receptor antagonist compared with control, but 17beta-estradiol did not strongly change LPS-stimulated cytokine secretion [196].

Most ER-dependent immune effects attributed to 17beta-estradiol in macrophages are thought to be mediated through ERalpha and not ERbeta [9, 197–199]. This has been widely supported by the findings of Ribas et al. [200]. Particularly, hematopoietic or myeloid-specific ERalpha deletion in mice displayed altered plasma adipokine and cytokine levels, glucose intolerance, insulin resistance, and increased AT mass. Moreover, LDL receptor-knockout mice transplanted with ERalpha null bone marrow presented a similar obese phenotype. Also, ERalpha was shown to mediate estrogens effects on inflammation attenuation, IL4-mediated induction of alternative activation, and maintenance of macrophage function [200]. In addition, GPER is also highly expressed in human macrophages [201].

Tissue macrophages can be classically (M1) or alternatively (M2) activated, expressing a profile of pro- and anti-inflammatory cytokines, respectively. Stimulation of macrophages with T_H_1 cytokines such as interferon gamma or LPS promotes classical activation of macrophages, which have a high inflammatory and bactericidal potential, characterized by the release of TNFalpha, IL6, and reactive oxygen species. However, T_H_2 cytokines, including IL4 and IL13, promote the alternative activation of macrophages, which have high antiparasitic capability and produce factors such as IL10, IL1 receptor antagonist, arginase, and transforming growth factor beta. M2 macrophages are also involved in remodeling (repair) of AT and lipid metabolism [189, 191]. Newly recruited AT macrophages display a more proinflammatory profile than resident AT macrophages in lean mice [202] and resident macrophages express genes characteristic of the M2 activation state [203, 204].

Macrophage differentiation strongly depends upon the local microenvironment. In activated macrophages, estrogens effects are primarily repressive, by inhibiting the expression of genes for cytokines or modulating other inflammatory mediators by the ER-dependent and/or nongenomic pathways in response to inflammatory signals [197, 205, 206]. Further, the majority of studies have only addressed the effect of estrogens on classically activated M1 cells and less is known about the effect on alternatively activated macrophages. The roles of estrogens in the macrophage-related inflammation are clearly complex. In mice, estrogens inhibit LPS-induced mouse homologue of MCP1 in peritoneal macrophages as well as IL6, IL1, and TNFalpha in splenic macrophages [207, 208]. Recent evidence suggests that activation of GPER participates in the downregulation of TNFalpha and IL in human macrophages [201]. The participation of NFkappaB activation has also been established since pretreatment of human macrophages with estradiol attenuates LPS-induced TNFalpha expression through the suppression of this transcription factor. Estradiol pretreatment abrogates the LPS-induced decrease in kappaB-Ras2, an inhibitor of NFkappaB signaling, via regulation of the two microRNAs let-7a and miR-125b [209].

## 5. Concluding Remarks

**Figure 4:**
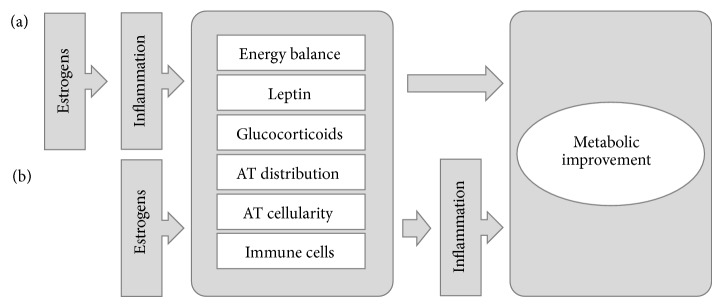
Estrogens effects on metabolic improvement may be cause (a) or consequence (b) of regulation of inflammation pathways. Further studies are needed to ascertain the relationship between estrogen signaling, inflammation, and metabolism. It is possible that the anti-inflammatory effects of estrogens, though their influences on processes like energy balance, leptin and glucocorticoid signaling, adipose tissue distribution and cellularity, and activity of immune cells, may culminate on metabolic improvement. However, estrogens have also been demonstrated to directly interfere with such processes, favoring improved inflammatory profile that results in overall metabolic amelioration. Additionally, the two hypotheses are not mutually exclusive. AT: adipose tissue.

**Figure 5:**
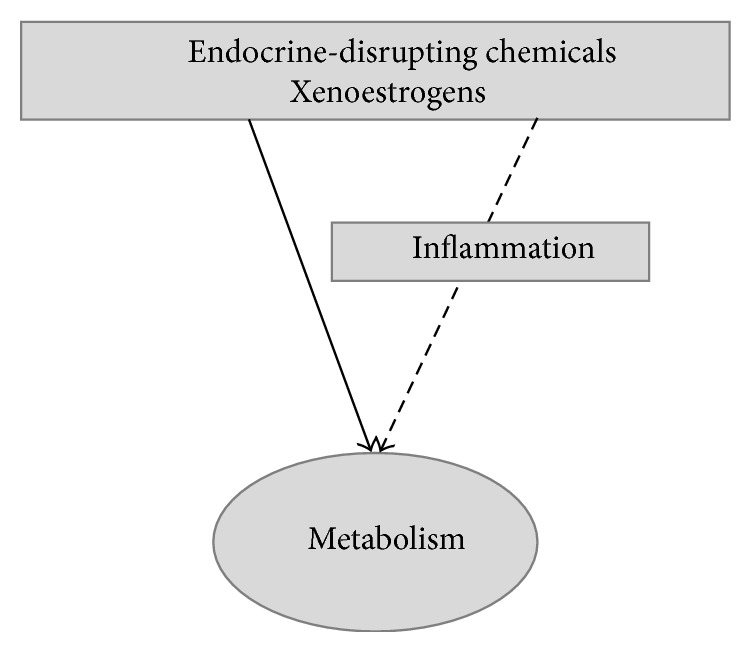
Effects of xenoestrogens on inflammation may mediate their actions on metabolism.

Evidence on the ability of estrogens to modulate metabolism-related inflammation is accumulating, although many questions remain unanswered. The independent influence of these hormones on each of these isolated areas of research is much more solid than the understanding that, through the modulation of inflammatory processes, estrogens are able to influence metabolic dysfunction. However, the relationship between esterogens and metabolism are reciprocal since the metabolic control favored by estrogens avoids the establishment of metabolic inflammation (Figure 4). The interdependence of both processes makes the studies of these matters elaborate as do the variation of estrogens' effects with timing of treatment, dose, type of estrogen used, physiological state of the organism, body composition, gender and availability of other hormones, and the multiple cellular pathways available for estrogen signaling. The recent demonstration of ubiquitous presence in living organism of xenobiotic molecules with endocrine disrupting properties with the ability to change estrogen signaling and largely impact on metabolism also deserves attention and may provide clues to the epidemic growth of obesity-related metabolic complications (Figure 5) [210–213]. Even so, there is general agreement that estrogens improve inflammation related to metabolic dysfunction and, although it seems evident that it acts indirectly through metabolic amelioration, the direct regulation of inflammation pathways is also documented. However, much work still needs to be performed in this respect to allow broadening the knowledge on estrogens' mechanisms of action and establishing the rationale for the development and use of estrogen signaling modulation as a therapeutic tool for metabolic improvement. Looking at the advances made on sister areas of research such as those related to the study of the inflammation-modulatory effects of estrogens on cardiovascular and neurodegenerative diseases and cancer [70, 72, 74] will possibly bring new additions to the mechanisms of estrogens' actions in metabolism.

## Figures and Tables

**Table 1:** Comparison between the effects of ovariectomy and estrogen reposition for metabolic inflammation-related processes. 11beta-HSD1, 11beta-hydroxysteroid dehydrogenase.

Ovariectomy	Reversal by estrogen treatment	References
Increases adipose tissue accumulation, mainly visceral, with increased adipocyte size	Yes Inhibition of key adipogenic genes, downregulation of lipoprotein lipase, and acetyl coenzyme A carboxylase and fatty acid synthase	[126, 187]

Increases lipoprotein lipase and lipid deposition in adipocytes	Yes	[177]

Increases food intake	Yes	[12, 50]

Reduces brain leptin sensitivity	Yes	[127]

Results in Insulin resistance and glucose intolerance	Yes	[127, 128, 187]

Promotes hepatic steatosis	Yes	[187]

Elevates low density lipoprotein, triglycerides, and fatty acids	Yes	[127]

Elevates proinflammatory markers	Yes However, increased transcription of inflammatory mediators induced by high-fat diet in the liver and visceral AT-interpreted as a dissociation between the metabolic and inflammatory effects	[127, 129, 187]

Increases hepatic 11beta-HSD1 activity and upregulates 11beta-HSD1 expression in visceral adipose tissue	Yes	[93, 214, 215]
Increases visceral compared to subcutaneous 11beta-HSD1	Increases subcutaneous compared to visceral 11beta-HSD1	

## Abbreviatons

AT: Adipose tissue
CREB: cAMP-related element binding
CREBH: cAMP responsive element-binding protein H
CPR: C-reactive protein
ER: Estrogen receptor
ERE: Estrogen response element
ERR: Estrogen-related receptor
GPER: G protein-coupled estrogen receptor
HRT: Hormone replacement therapy
IkappaB: Inhibitor of kappaB
IKK: Inhibitor of kappaB kinase
IL: Interleukin
JNK: c-jun N-terminal kinase
LDL: Low density lipoprotein
MAPK: Mitogen-activated protein kinase
M1: Classically activated macrophages
M2: Alternatively activated macrophages
MCP1: Monocyte chemoattractant protein 1
NAFLD: Nonalcoholic fatty liver disease
NFkappaB: Nuclear factor kappaB
NO: Nitric oxide
PAI1: Plasminogen activator inhibitor 1
PGC1: Peroxisome proliferator-activated receptor gamma coactivator 1
PI3K: Phosphoinositide 3-kinase
PPAR: Peroxisome proliferator-activated receptor
TF: Transcription factor
TLR: Toll-like receptor
TNF: Tumor necrosis factor
UCP: Uncoupling protein
17Beta-HSD: 17Beta-hydroxysteroid dehydrogenase.
